# Robust Type-specific Hemisynapses Induced by Artificial Dendrites

**DOI:** 10.1038/srep24210

**Published:** 2016-04-13

**Authors:** Eun Joong Kim, Chang Su Jeon, Soo Youn Lee, Inseong Hwang, Taek Dong Chung

**Affiliations:** 1 Department of Chemistry, Seoul National University, Seoul, 08826, Korea

## Abstract

Type-specificity of synapses, excitatory and inhibitory, regulates information process in neural networks via chemical neurotransmitters. To lay a foundation of synapse-based neural interfaces, artificial dendrites are generated by covering abiotic substrata with ectodomains of type-specific synaptogenic proteins that are C-terminally tagged with biotinylated fluorescent proteins. The excitatory artificial synapses displaying engineered ectodomains of postsynaptic neuroligin-1 (NL1) induce the formation of excitatory presynapses with mixed culture of neurons in various developmental stages, while the inhibitory artificial dendrites displaying engineered NL2 and Slitrk3 induce inhibitory presynapses only with mature neurons. By contrast, if the artificial dendrites are applied to the axonal components of micropatterned neurons, correctly-matched synaptic specificity emerges regardless of the neuronal developmental stages. The hemisynapses retain their initially established type-specificity during neuronal development and maintain their synaptic strength provided live neurons, implying the possibility of durable synapse-based biointerfaces.

In biological systems, the connection between neurons is dynamically secured by a chemical synapse whose gap is filled with a variety of trans-synaptic cell adhesion molecules (CAMs) that specify the excitatory and inhibitory properties^1^. At the presynapses, the electrical signals, called action potentials (APs), are converted into chemical signals mediated by neurotransmitters that either excite or inhibit postsynaptic APs depending upon the types of the chemical synapses. Consequently, a sophisticated regulation of excitatory and inhibitory signals is crucial for the constitution of functional neuronal networks^2^. Such complexity of brain nervous system makes it difficult to reconstitute broken neuronal pathways or damaged part of the brain^3^,^4^.

One of the most striking features in synaptogenesis is that neurons can form ‘hemisynapses’ with a non-biological partner whose surface is covered with functional synaptic CAMs^5^,^6^,^7^. Such hemisynapses garner much attention because they can secure the connection between the two heterologous systems with defined geometry^8^,^9^,^10^. However, there has been lack of information about the dependency of neuronal morphology on synaptic specificity, the durability of excitatory and inhibitory hemisynapses, and the methodology of synaptic CAM engineering for the generation of robust artificial dendrites that enable the formation of type-specific hemisynapses.

Among the trans-synaptic interactions between the synaptic CAMs, the adhesion between postsynaptic neuroligins (NLs) and presynaptic neurexins (Nrxs) plays pivotal roles in synapse formation, differentiation and validation^11^. The excitatory (glutamatergic) presynaptic differentiation is induced by NL1, 3 and 4, and the inhibitory (GABAergic) by NL2^12^,^13^,^14^,^15^. While many other synaptic CAMs and their roles are rapidly emerging, only Slitrk3–PTPδ pair has shown an unprecedented specificity to inhibitory synapses^16^,^17^,^18^.

Herein, we reconstituted durable inhibitory and excitatory hemisynapses with spatiotemporal regulation using type-specific artificial dendrites that display modularly-tagged ectodomains of postsynaptic CAMs, such as NL1, NL2, and Slitrk3. We found that in random culture of hippocampal neurons the artificial dendrites can form correctly-matched hemisynapses with fully developed neurons, whereas with immature neurons only excitatory hemisynapses. Remarkably, in micropatterned culture of neurons mimicking aligned neural networks in the hippocampus, the artificial dendrites can form correctly-matched hemisynapses with isolated axonal components of immature neurons. Furthermore, the synaptic strength and the type-specificity of the hemisynapses, once established, remained unchanged as long as live cells are provided.

## Results and Discussion

### Construction of modularly-tagged synaptic CAM ectodomains

To generate type-specific artificial dendrites, ectodomains of NL1 (48–639), NL2 (15–614), and Slitrk3 (28-649) were excerpted from their full sequences. NL1 ectodomain was determined by the minimally secreted functional domain^19^ and the crystal structure that reveals Leu636 as the end of the α-helix required for NL1 dimerization^20^,^21^ that induces presynaptic differentiation via clustering of presynaptic neurexins (Nrxs). Ectodomain of NL2 (15–614) was determined by sequence comparison with NL1. The Slitrk3 ectodomain contains two leucine-rich repeat domains (LRR1 and LRR2) followed by several extra C-terminal residues.

**Figure 1:**
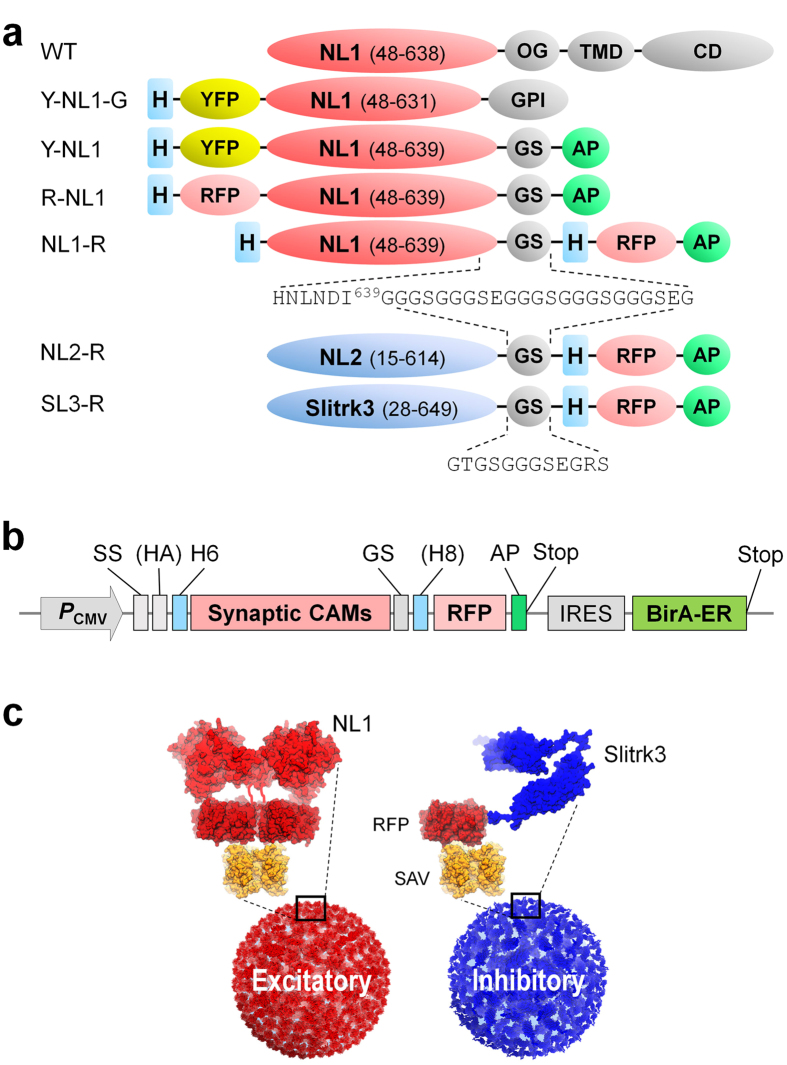
(**a**) Comparison of domain structures of engineered ectodomain of postsynaptic cell adhesion molecules. WT, wild type; YFP, yellow fluorescent protein; RFP, red fluorescent protein (TagRFP-T); H, hexa- or octa-His tag; OG, O-glycosylation rich domain; TMD, transmembrane domain; CD, cytoplasmic domain; GPI, glycosylphosphatidylinositol anchoring motif; GS, glycine-serine linker; AP, biotin acceptor peptide (AviTag). (**b**) Structure of optimized expression vector. Bicistronic expression of BirA for mass production of *in vivo* biotinylated postsynaptic CAMs. SS, signal sequence; HA, hemagglutinin affinity tag; H6, hexa-His tag; H8, octa-His tag; IRES, internal ribosome entry site. The SS, HA and H6 can vary depending upon the type of the postsynaptic CAMs. (**c**) Schematic structure of excitatory and inhibitory artificial dendrites displaying C-terminally tagged ectodomains of postsynaptic NL1 homodimer (NL1-R) and Slitrk3 monomer (SL3-R). The *in vivo* biotinylated fluorescence tagging module enables oriented immobilization of the ectodomains on streptavidin (SAV) surface. The structure of NL1 was modelled after NL1 (PDB ID, 3BIX)^20^ while the structure of Slitrk3 (27–627 residues) was predicted using Slitrk1 (PDB ID, 4RCW)^31^ as a template by the RaptorX server^32^, showing two LRR1 and LRR2 domains.

To facilitate massive production, purification, quantification, and localization of the ectodomains, we designed a tagging module that can be generally applied to virtually all kinds of synaptic CAM ectodomains. We began by engineering the NL1 ectodomain by placing YFP or RFP either at the N- or C-terminus of the protein and a biotin acceptor peptide (AP, also called AviTag)^22^ at the end of the expression cassette to utilize biotin-streptavidin (SAV) interaction for immobilization of the protein (Figs 1 and S1a,b). The *in vivo* biotinylation was further simplified by bicistronic expression of endoplasmic reticulum-specific bacterial biotin ligase (BirA-ER) (Fig. 1b).

### C-terminal fusion is optimal for NL1 ectodomain function *in vitro*

**Figure 2:**
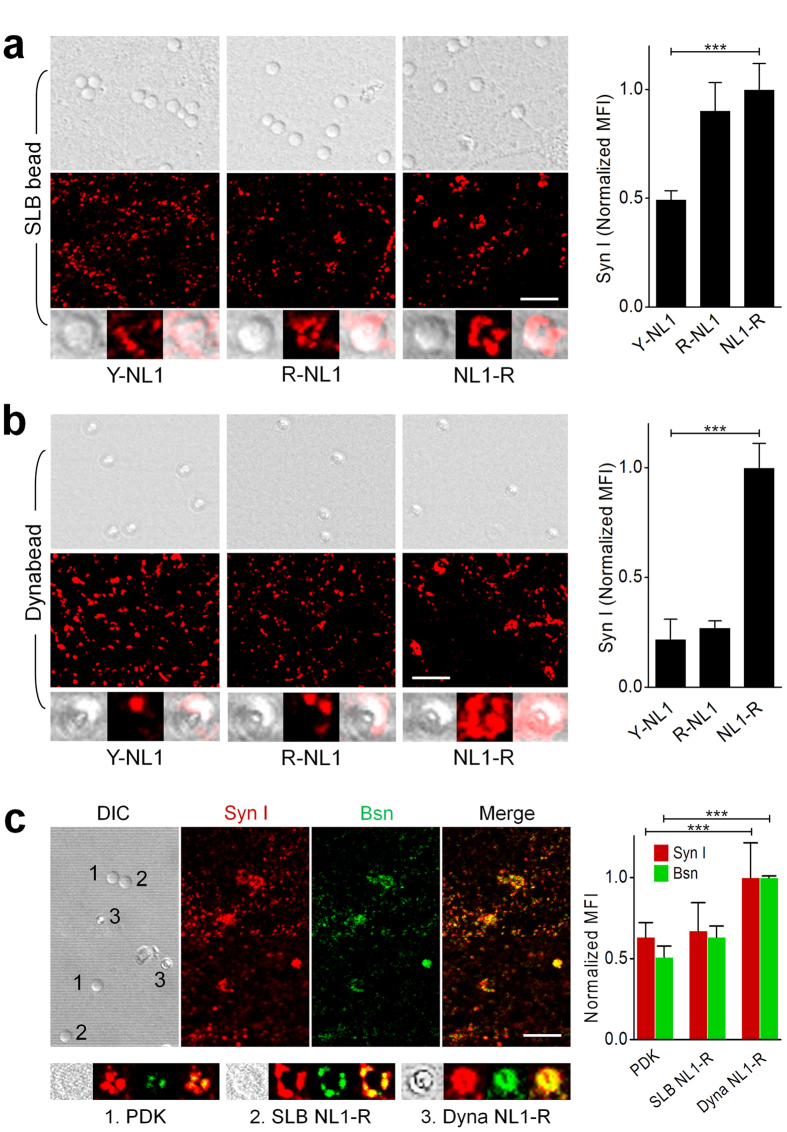
NL1-R induces presynaptic differentiation independently of SLB membrane. (**a–c**) Immunocytochemistry (ICC) analysis for synaptic marker proteins in cultured hippocampal neuron. (**a**) NL1-R has the strongest synaptogenic activity both on SLB membrane beads and SAV Dynabeads when incubated with DIV14 hippocampal neuron culture for 2 d. (**b**) NL1-R shows the most prominent synaptogenic activity when immobilized on microbeads without SLB membrane. Synapsin I (Syn I) and bassoon (Bsn) aggregation were induced by PDK-coated silica beads, NL1-R on SLB, and NL1-R on SAV Dynabeads. MFI = mean fluorescence intensity. Scale bars = 10 μm. ^∗∗∗^p < 0.001.

Although the previously reported excitatory hemisynapses were induced by NL1 ectodomain docked on obligate supported lipid bilayer (SLB) membrane^5^,^6^, obviating the lipid layer should simplify the fabrication process of the artificial dendrites and greatly extend the types of surface on which synaptic proteins are immobilized. In addition, we can minimize a hindrance to communication, if possible, between neurons and the artificial dendrites. When we compared the synaptogenic activity of the N- or C-terminally-tagged NL1 ectodomains immobilized on SAV-coated microbeads (Dynabeads M-280 Streptavidin, 2.8 μm in diameter) and silica microbeads (5 μm in diameter) covered with SAV-loaded fluidic SLB membrane (Fig. S1c), only C-terminally tagged NL1-R immobilized without lipid layer showed the most prominent synaptogenic activity by showing aggregated synapsin I, a general synapse marker protein (Fig. 2). The synaptogenic potential of NL1-R was greater than the one of poly-D-lysine (PDK) that is also known to induce the formation of synaptic boutons with mature neurons^10^,^23^,^24^. We then applied the same tagging module to the inhibitory NL2 and Slitrk3 ectodomains to establish a complementary type-specificity, generating NL2-R and SL3-R (Fig. 1a).

### NL1-R forms durable glutamatergic presynaptic boutons independently of developmental stages of neurons

**Figure 3:**
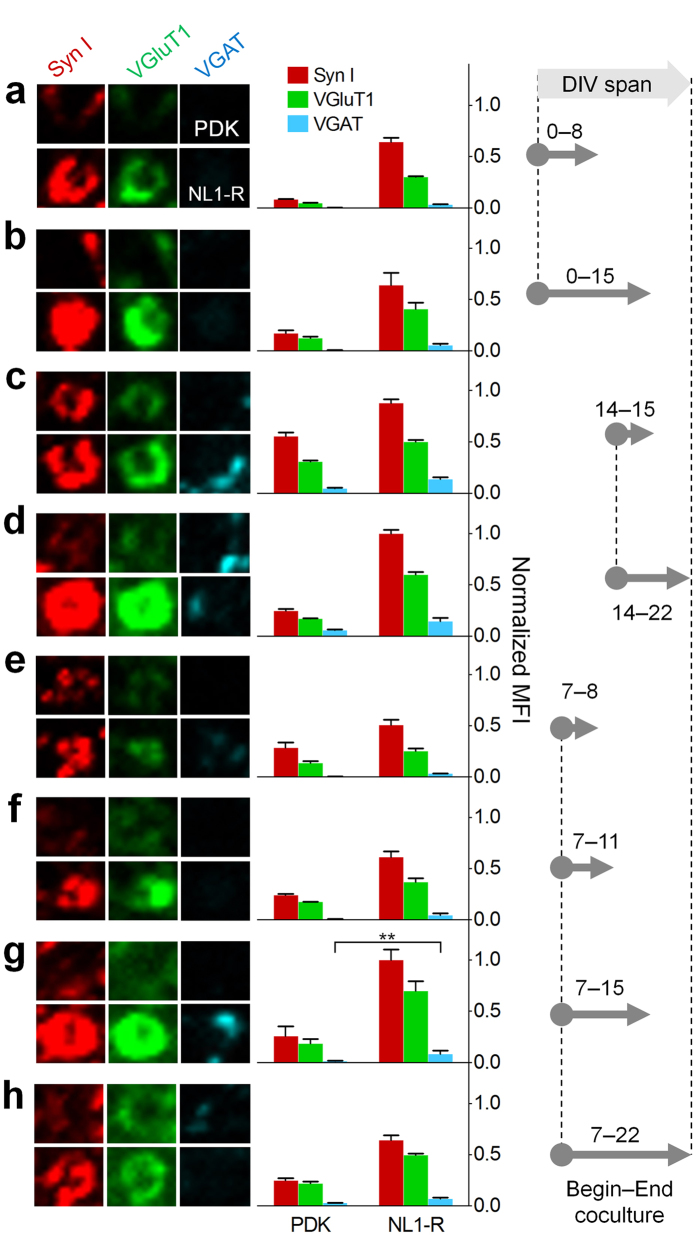
NL1-R induces the formation of glutamatergic presynaptic boutons regardless of the developmental stages of cultured neurons and the incubation period. (**a–h**) ICC images for PDK-induced hemisynapses are aligned on upper lines. The cells were doubly stained either by anti-synapsin I (Syn I, red), VGluT1 (green), and anti-VGAT (cyan). The PDK and NL1-R beads were seeded on DIV0 and incubated for 8 d (**a**) and 15 d (**b**). The beads seeded on DIV14 were incubated for 1 d (**c**) and 8 d (**d**). When seeded on DIV7, the incubation was continued for 1 d (**e**), 4 d (**f**), 8 d (**g**), and 15 d (**h**). ^∗∗^p < 0.01; others p < 0.001 between the same marker proteins of PDK and NL1-R.

The PDK-coated beads serve as a synaptogenic substrates only when they are introduced to dissociated hippocampal cells after a certain developmental stage (at least DIV7)^10^. The PDK-induced hemisynapses, instructed by F-actin reorganization and heparan sulfate proteoglycans (HSPGs), increased after 24 h incubation and could be observed up to 72 h^10^. Unfortunately, they lack synapse specificity, inducing the formation of both excitatory and inhibitory synaptic boutons. Postsynaptic NL1, on the other hand, is a negatively charged protein that specifically binds to β-Nrx expressed on outgrowing axonal surface, thereby regulating the synapse specificity. However, synaptic CAMs can induce the formation of mismatched synapses between glutamatergic and GABAergic synaptic terminals in the early developmental stages^23^,^24^,^25^. We thus examined the synaptic specificity of the NL1-coated artificial dendrites by seeding them to mixed culture of neurons in various developmental stages. When we seeded the PDK and NL1-R beads from the beginning of the neuron culture (DIV0) and incubated up to DIV8 and 15, we found that in both durations only the NL1-R-coated beads successfully induced excitatory presynaptic differentiation, showing immunoreactivity toward synapsin I and VGluT1, an excitatory synapse marker protein, but not toward vesicular GABA transporter (VGAT), an inhibitory synapse marker protein (Figs 3a,b and S2a,b). As expected, PDK could induce presynaptic differentiation when they met only with the fully developed cells (DIV14), showing slightly weaker hemisynapses than NL-1R as shown in (Figs 2c,3c and S2c). When we elongated the incubation up to 8 d, however, the majority of the synaptic boutons generated by the PDK beads diminished while the ones induced by the NL1-R beads increased (Figs 3d and S2d). When the beads were seeded on DIV7, only NL1-R exerted synaptogenic activity upon incubation for 8 d (Figs 3e–h and S2e–h), similar to the result for NL1-R seeded on DIV14 (see Figs 3c and S2c).

The polybasic molecules such as PDK, if applied to developed neurons, can satisfy the eager for the synaptogenesis of outgrowing axons by allowing hemisynapse formation. It seems, however, that the factors sustaining hemisynapses become accustomed to the PDK molecules and eventually lose their synaptogenic potential upon extended period of coincubation. In comparison, the NL1-R artificial dendrites not only induced excitatory hemisynapses with neurons at any developmental stages, but also strengthened and maintained the excitatory specificity of the synapses as long as live neurons are provided.

### NL2-R and SL3-R forms inhibitory presynaptic boutons only with mature neurons

**Figure 4:**
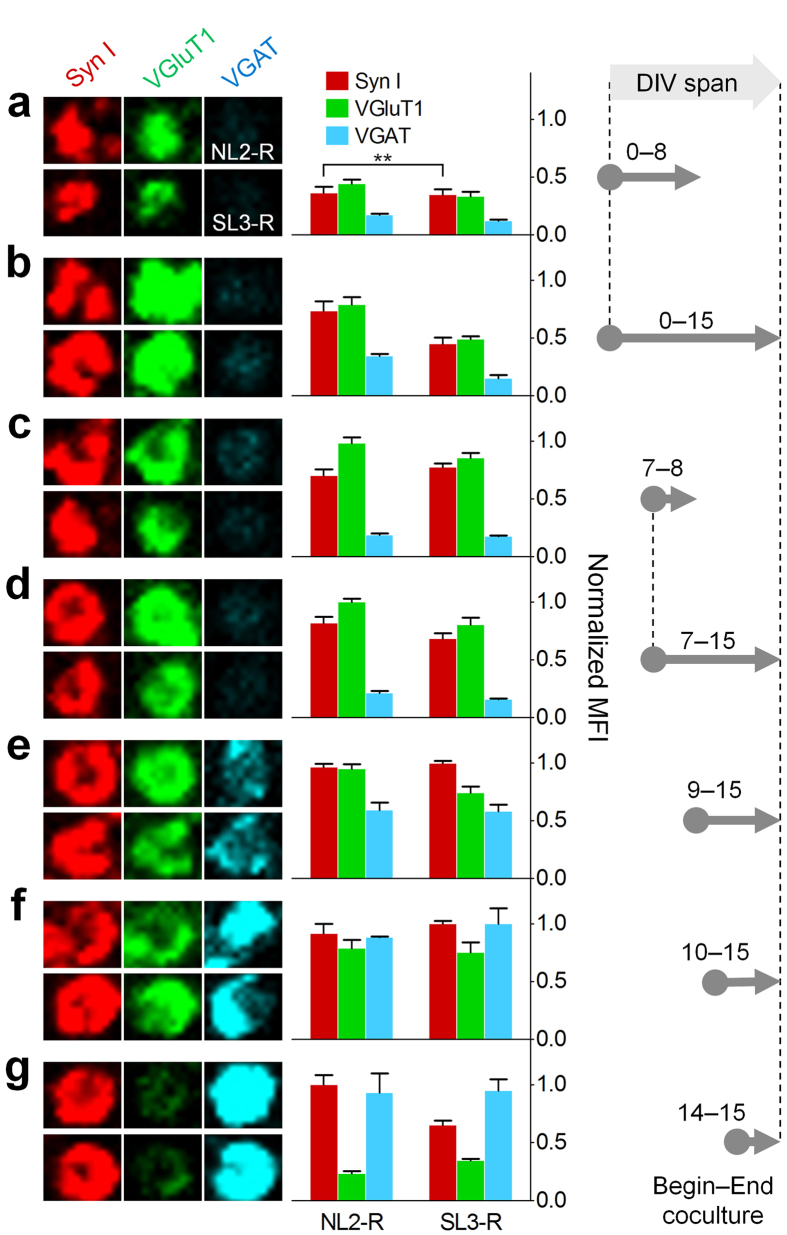
NL2-R and SL3-R induce the formation of inhibitory hemisynapses only with mature neurons. The NL2-R and SL3-R beads were seeded and analysed for the synaptogenesis as in the case of NL1-R. (**a–g**) ICC images for NL2-R-induced hemisynapses are aligned on upper lines. (**a–d**) Only excitatory presynaptic differentiations appeared during the early neuronal development stages, which lasted until DIV15. Correctly matched synapses began to appear on DIV9 when the glutamatergic specificity is still dominant (**e**). The fully inhibitory hemisynapses could be formed only when the artificial dendritic beads are in contact with mature neurons (DIV14) (**g**). ^∗∗^p < 0.01; others, p < 0.001 between the same marker proteins of NL2-R and SL3-R.

We next applied the NL2-R- and SL3-R-coated inhibitory artificial dendrites on neuron cultures in various developmental stages to compare the timing of the instruction of inhibitory hemisynapses. When we added NL2-R or SL3-R beads to immature neuronal cells (DIV0 and 7), we could observe only excitatory presynaptic differentiation regardless of the coculture duration (Figs 4a–d and S3a–d,h–k). The inhibitory synaptic specificity appeared when the coculture started on DIV9 and was fully restored on DIV14 (Figs 4e–g and S3e–g,l–n). Thus, it was the timing of the encounter with artificial dendrites that determined the inverted synaptic specificity of growing neurons.

In the developing hippocampal neurons, GABAergic synapses are formed before glutamatergic synapses^26^. In the dissociated hippocampal neurons, however, the formation of glutamatergic synapses typically precedes the one of GABAergic synapses^26^,^27^, where the mismatched synapses form frequently. This mismatch diminishes as neurons mature, probably promoted by the regulated expression of type-specific synaptic CAMs or by increased transmitter activity between matched synaptic machineries^26^ including a signalling for proteolytic cleavage of synaptic adhesion proteins for sculpting of the mismatched synapses^28^. By contrast, our abiotic artificial dendrites are immune to neurotransmitter signals and proteolytic cleavage, not allowing correction of initially formed mismatched specificity. It is possible that the NL1-R-induced glutamatergic hemisynapses found in the immature hippocampal neurons (see Fig. 3) originate in part from a simple mismatch independent of the type-specific synaptic CAMs. Thus, in classical culture of hippocampal neurons, the synaptic specificity defined by the synaptic vesicle proteins is determined not by the synaptic CAMs but by the degree of neuronal development.

### Axonal compartmentalization allows the formation of type-specific synapse networks with artificial dendrites

**Figure 5:**
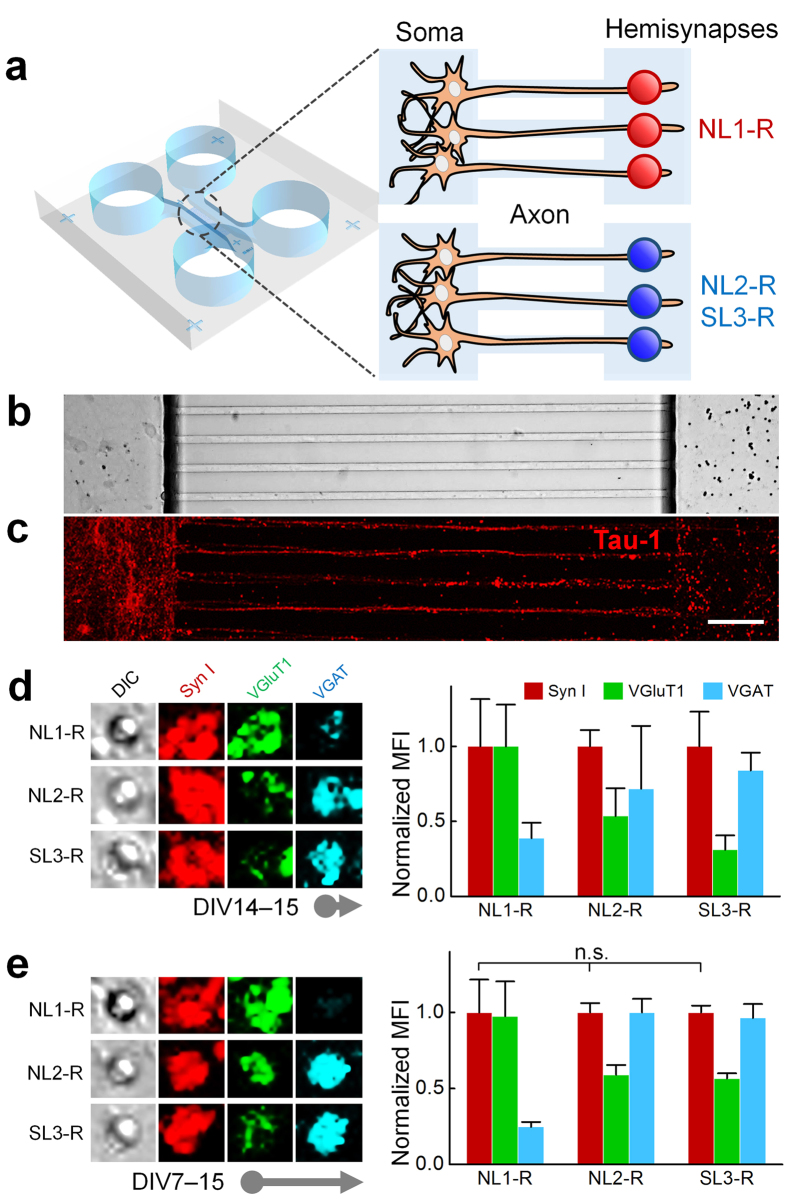
Axons are compartmentalized to mimic patterned neural networks found in hippocampus. (**a**) Schematics showing isolated axons in contact with excitatory (NL1-R) and inhibitory (NL2-R and SL3-R) artificial dendrites. Light image (**b**) and Tau-1 staining (**c**) shows aligned axon bundles with seeded neurons on the left and the artificial dendrites on the right. Scale bar = 20 μm. (**d**) The neuronal cells and the NL1-R beads were incubated for 1 d from DIV14 (**d**) and for 8d from DIV7 (**e**) followed by the ICC analysis. n.s., not significant; others, p < 0.001 between the same marker proteins of NL1-R, NL2-R, and SL3-R.

In the brain, neurons manifest a polarized subcellular structure and complex geometry through which the formation of synapses by the synaptic CAMs is chronologically and geometrically controlled. In order to address the functional importance of the subcellular synaptic specificity, we reconstituted spatial compartmentalization using microfluidic culture chamber that enables isolation of axons from somata in an analogous fashion to the patterned hippocampal neural networks^29^,^30^. The directional growth of neurons provides a homogeneous population of axons with which the postsynaptic CAM-covered solid substrates can form hemisynapses without intervening endogenous dendrito- and somato-axonic synapses (Fig. 5a–c).

About one week after seeding the neuronal cells in the left somal side of the chamber, axons escaped the microchannels to reach the right compartment of the chamber and covered about two-thirds of the field on DIV14. After brief verification of synaptogenic activity of NL1-R beads (Fig. S4), we applied artificial dendrites displaying NL1-R, NL2-R, and SL3-R to the axonal compartments and compared the developmental pattern of the synaptic specificity. As expected, in the axonal compartment of adult neurons the artificial dendrites exerted correctly matched synaptic specificity, similar to the results obtained from random-cultured mature neurons (Figs 4d and S5a; also see Figs 2 and 3). Surprisingly, when we seeded the synaptic CAM beads on DIV7 and incubated for 8 d, there was no inversion of synaptic specificity caused by inhibitory NL2-R and SL3-R beads (Figs 4e and S5b), showing similar patterns of synaptic puncta between the beads near and far from the microchannels (Fig. S6). Thus, in order to induce correctly matched hemisynapses, the artificial dendrites appear to require isolated axonal components of cultured neurons.

### NL1-R-induced presynaptic boutons are glutamatergically active

**Figure 6:**
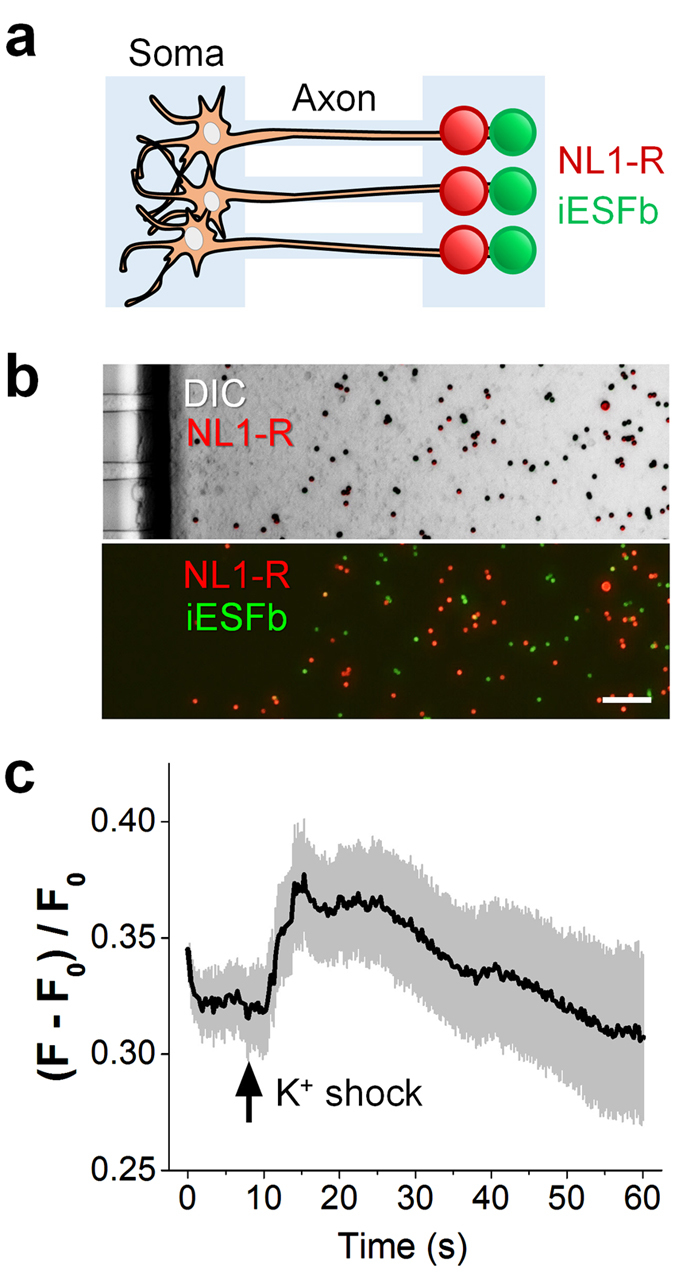
Excitatory hemisynapses release glutamate neurotransmitter. (**a**) Schematics of the measurement of glutamate spillover using engineered glutamate sensor iESFb. (**b**) NL1-R artificial dendrites were added to the axonal compartment of DIV14 cells. After incubation for 2 d, the iESFb beads were spread to the same side. Scale bar = 10 μm. (**c**) The neurons were stimulated by potassium shock on the somal compartment. When compared with bare SAV beads, the iESFb beads near the artificial dendrites exhibited enhanced fluorescence due to the glutamate spillover.

Having established the polarized artificial synaptic interfaces, we wanted to ensure the synaptic functionality of the hemisynapses to genuinely mimic the directional flow of neuronal information via neurotransmitters. We thus engineered the intensity-based glutamate-sensing fluorescent reporter (iGluSnFR)^33^ to directly measure the rapid glutamate transients. The engineered iGluSnFR, dubbed iESFb, shares C-terminal tagging module with our synaptic CAM ectodomains and has the sensitivity enough for our experiments (will be reported elsewhere). We placed the NL1-R beads in the axonal side of the culture chamber on 14 DIV, allowing two days of hemisynapse formation before spreading the freshly prepared iESFb beads (Fig. 6a,b). Upon potassium shock in the somal side of the chamber, we could observe an increase in green fluorescence of iESFb near NL1-R beads in comparison to SAV beads without NL1-R, indicating that the hemisynapses possess type-specific neurotransmitter activity (Fig. 6c).

Abiotic solid substrata displaying engineered ectodomains of postsynaptic CAMs can serve as artificial dendrites, inducing the formation of durable excitatory and inhibitory hemisynapses with spatiotemporal regulation. The ectodomains of NL1, NL2, and Slitrk3 allow C-terminal tagging for optical tracking and surface immobilization that dispenses with lipid bilayers for its synaptogenic activity. The modular C-terminal tags may change ectodomains of virtually any pre- and postsynaptic CAMs that have functional extracellular domains. Notably, the artificial dendrites could instruct the formation of inhibitory hemisynapses with dissociated culture of well-developed neurons and with isolated axonal component of micropatterned neurons even in their early developing stages. Once determined, either the matched or mismatched synaptic specificity of hemisynapses remained unchanged during the extended neuronal development. Such robust hemisynapses should bode well for a fundamental study on information process of neural networks in the brain and novel type of synapse-based biointerfaces where signals are transduced via synaptic boutons with defined synaptic specificity and spatial geometry.

## Materials and Methods

### Reagents

Plasmids encoding cholinesterase-like domain (CLD) of neuroligin-1 (NL1) followed by GPI anchoring motif (pNICE-HA-H6-NL1-GPI) and full length NL1 and NL2 (pNICE-HA-H6-NL1 and pNICE-HA-NL2) were a generous gift from Peter Scheiffele (University of Basel). A plasmid carrying EYFP-tagged full length NL1 (pNICE-YFP-NL1) was a kind gift from Ann Marie Craig (University of British Columbia). A plasmid encoding full length Slitrk3, pGW1-Slitrk3, was a kind gift from Jaewon Ko (Yonsei University). A plasmid expressing TagRFP-T (pcDNA3-TagRFP-T) was kindly provided by Roger Y. Tsien (University of California). BirA expressing plasmids pDisplay-BirA-ER (Addgene plasmid #20856) and pET21a-BirA (Addgene plasmid #20857) were provided by Alice Ting. pAd:CMV-rtTA-IRES-BirA was a gift from William Pu (Addgene plasmid #31375). Optical glutamate sensor encoding pCMV(MinDis).iGluSnFR was a gift from Loren Looger (Addgene plasmid #41732). Mammalian vector pDisplay, Flp-In system, and Dynabeads® M-280 Streptavidin were obtained from Life Technologies (Grand Island, NY). Poly-D-lysine (PDK, Mw 70,000–150,000, P0899) was from Sigma (Seoul, Korea). Silica beads (2.56 and 5 μm in diameter) were from Bangs Laboratories, Inc. (Fishers, IN). Egg phosphatidylcholine (PC) and 1,2-dipalmitoyl-sn-glycero-3-phosphoethanolamine-*N*-(cap-biotinyl) (sodium salt) (Biotin-Cap-PE) were from Avanti Polar Lipids (Alabaster, Al). Goat NL1 polyclonal antibody (sc-14084) was from Santa Cruz Biotechnology, Inc. (Santa Cruz, CA). Rabbit synapsin I polyclonal antibody (AB1543), guinea pig VGluT1 polyclonal anitibody (AB5905), mouse tau monoclonal antibody (MAB3420), and rabbit VGAT polyclonal antibody (AB5062P) were from Merck Millipore (Billerica, MA). Mouse bassoon monoclonal antibody (ab82958) was from Abcam (Cambridge, UK). Mouse synapsin I monoclonal antibody (106 001) and rabbit VGluT1 polyclonal antibody (135 303) were from Synaptic Systems (Goettingen, Germany/DE).

### Molecular biology

To make protein Y-NL1-G, the plasmid pNICE-YFP-NL1 was cut by KpnI and NotI to replace the predicted OG, TMD, and CD with the GPI anchor excised from pNICE-HA-H6-NL1-GPI with the same endonucleases. To make protein Y-NL1, the GPI portion of the pNICE-YFP-NL1-GPI plasmid was replaced with GS-AP, a GS-linker (HLHNLDI^639^ GGGSGGGSEGGGSGGGSGGGSEG) fused with a 14-mer AP (AviTag, GLNDIFEAQKIEWHE) prepared by the PCR-based staggered extension (StEP) using primers N38, N39, N40 and N41 followed by KpnI and NotI treatment. To aid purification, His×8 encoding primers, N43 and N44, were annealed and introduced in front of the YFP sequence using a single PvuI site, yielding pNICE-H8-YFP-NL1-AP (encoding Y-NL1). To obtain protein R-NL1, TagRFP-T was PCR amplified from the pcDNA3-TagRFP-T plasmid using primers N48 and N49, cut by PvuI and SalI, and ligated with pNICE-H8-YFP-NL1-AP that had been digested with the same restriction enzymes, resulting in pNICE-H8-RFP-NL1-AP. To produce protein NL1-R, we first replaced the GPI tag of pNICE-HA-H6-NL1-GPI with a new GS linker containing additional cloning sites, PvuI and SalI, and AP tag (PHLHNLDI^639^ GGGSGGGSEGGGSGGGSGGGSEG−RS^(PvuI)^GVD^(SalI)^−(AP)). The GS linker was synthesized using primers N38, N39, N50 and N41 by StEP, cut by KpnI and NotI, and ligated with pNICE-HA-H6-NL1-GPI that had been cut by KpnI and NotI, yielding pNICE-HA-H6-NL1-GS-PS-AP. The TagRFP-T PCR product obtained from primers N48 and N49 and pNICE-HA-H6-NL1-GS-PS-AP were then digested with PvuI and SalI, followed by the ligation giving pNICE-HA-H6-NL1-H8-RFP-AP (encoding NL1-R). To make NL2-R, ectodomain of NL2 was amplified from pNICE-HA-NL2 using primers N55 and N56. The GS linker was PCR amplified from pNICE-HA-H6-NL1-H8-RFP-AP using primers N103 and N104. The pNICE-HA-H6-NL1-H8-RFP-AP was but by BamH I and SalI and served as a vector for three-piece Gibson assembly. To make SL3-R, ectodomain of Slitrk3 was copied from pGW1-Slitrk3 using primers N83 and N74-2 and cut by HindIII and KpnI. The GS linker fused with H8-RFP, GT^(KpnI)^GSGGGSEGGGSGGGSGGGSEG-RS^(PvuI)^−(H8-RFP)−VD^(SalI)^−(AP), was PCR-amplified from pNICE-HA-H6-NL1-H8-RFP-AP using N38-2 and N41 and cut by KpnI and NotI. The pNICE-HA-H6-NL1-GS-AP was cut by HindIII and NotI and served as a vector for three-piece ligation, finally giving pNICE-SL3-H8-RFP-AP encoding SL3-R. To screen linker sequences, pNICE-SL3-H8-RFP-AP was cleaved by KpnI and PvuI where other linkers with various lengths were inserted. The active SL-R was obtained using annealed primers N106 and N107, yielding GT^(KpnI)^GSGGGSEGRS^(PvuI)^−(H8-RFP)−VD^(SalI)^−(AP). To make a bicistronic expression vector that allows a simultaneous expression of BirA-ER with the postsynaptic CAMs, the internal ribosome entry site (IRES) followed by BirA-ER gene was introduced. The pNICE-(HA-H6)-CAM-H8-RFP-AP (CAM = ectodomain of NL1, NL2, or Slitrk3) were cleaved by NotI to insert IRES-BirA that was amplified from pAd:CMV-rtTA-IRES-BirA using primers N86 and N87 and cut by NotI, generating pNICE-CAM-RAP-BirA after ligation. Bacterial expression and purification of bacterial BirA biotin ligase was conducted using pET21a-BirA plasmid. The major primers used in this study are listed in the Table S1.

### Stable cell lines

For the initial screening of synaptogenic activity of NL1 ectodomains, NL1-encoding plasmid DNA (pNICE-H8-YFP-NL1-AP, pNICE-H8-RFP-NL1-AP, and pNICE-HA-H6-NL1-H8-RFP-AP) (24 μg of each) was added to the Opti-MEM I Reduced Serum Media (1.5 mL). Likewise, 1.0 mg/mL PEI (60 μl) solution was added to the Opti-MEM solution (1.5 mL). After 5 min incubation at 25 °C, the two solutions were mixed at room temperature for 30 min and added to HEK293 cells grown to ~20% confluence in a culture dish of 10 cm diameter at 37 °C. The DMEM medium was replaced after 4 h incubation. After three days, the cells were treated with G418 (final conc. 0.8 mg/mL). The G418 treatment was repeated with fresh medium after two days. After two weeks, single colonies with brightest fluorescence signals were picked and seeded on a 24-well plate. Among them, the best fluorescent colonies were repeatedly selected until only one colony was left and seeded on a culture dish of 10 cm diameter for the subsequent passage. The established stable cell line was kept in DMEM medium containing G418 (100 μg/mL).

### Preparation of biotinylated synaptic CAMs

Plasmid pDisplay-BirA-ER (24 μg) in Opti-MEM solution (1.5 mL) was mixed with Opti-MEM solution (1.56 mL containing 60 μg of PEI) for 20 min at 25 °C. The mixture was added to the established HEK293-H stable cell lines at ~20% confluence in a culture dish of 10 cm diameter. After 4 h incubation, the DMEM medium was replaced with a fresh one containing G418 (100 μg/mL) and biotin (10 μM). The *in vivo* biotinylated synaptic CAM was allowed to secrete into culture medium for three days at 37 °C. Then, the medium (10 mL) was saved and the whole cells were transferred to a culture dish of 15 cm diameter and filled with 30 mL of DMEM containing G418 (100 μg/mL) and biotin (10 μM). After another three days, the culture media were combined and subjected to column purification using 50% Ni-NTA resin (2 mL) according to the manufacturer’s protocol. The three elution fractions (1 mL) were subjected to fluorescence analysis using Synergy Mx fluorescence microplate reader (BioTek, Seoul, Korea).

For *in vitro* biotinylation, the stable cell line was grown without BirA transfection. To the column elution fraction (1 mL) showing the highest fluorescence signal was added MgCl_2_ (5 mM), ATP (1 mM), biotin (0.1 mM), and BirA enzyme (30 nM) as final concentration and shake incubated for 2 h at 37 °C. The level of *in vivo* and *in vitro* biotinylation was analysed via western blot using SAV-HRP or using goat or mouse anti-NL1 antibody and HRP-conjugated secondary antibody. The purity of NL1 was analysed by SDS-PAGE with silver staining.

### Preparation of PDK microbeads

Silica microbeads (2.56 and 5.06 μm in diameter, 3 × 10^5^ beads) were incubated with PDK solution (0.5 mg/mL in DPBS) overnight at room temperature, washed three times with DPBS, and spread on cultured neurons per condition. For the coincubation with the artificial dendrites, 0.5 × 10^5^ beads were spread. Otherwise, 1.0 × 10^5^ beads were used to examine the formation of the PDK hemisynapse.

### Quantification of synaptic CAMs on SLB microbeads

The synaptic CAM-SLB microbeads (7.5 × 10^4^ beads) solution was micro-centrifuged for 1 min and the supernatant was removed. To this was added 50 μl of TBS/2% Triton X-100 followed by brief vortexing. After 10 min incubation, the mixture was micro-centrifuged for 1 min and the supernatant was transferred to a 96-well plated to read fluorescence by Synergy Mx fluorescence microplate reader (BioTek, Seoul, Korea). The amount of the proteins was calculated by applying conversion factor, 19,000 MFI/μL for NL1-R, obtained by SDS-PAGE using BSA standard and relative molecular mass (*M*_r_) of the protein, 135,000.

### Neuronal cell culture

Primary hippocampal neurons were obtained from Sprague-Dawley rat embryos at day 18 of gestation (E18). Briefly, hippocampus dissected from E18 rat embryos were rinsed with HBSS, and then incubated with papain and DNase with 60 rpm for 30 min at 37 °C. After sequential rinsing with solution of 10% and 5% FBS in HBSS, individual single cells were mechanically isolated by trituration 10 times in 2 mL HBSS containing DNase with silanized Pasteur pipette of which tip was barely polished with fire. The cell suspension was diluted to density of 2 × 10^5^ cells/mL with plating media containing MEM supplemented with 0.6% (w/v) glucose, 10 mM sodium pyruvate, 1 mg/mL FBS, and 1% penicillin-streptomycin. Then, the cell-media solution plated on the PDK-coated glasses placed in Petri dish. After 3 h the cell culture media was exchanged with B27-supplemented Neurobasal Media containing 2 mM Glutamax. Cultures were maintained in an incubator at 37 °C and 5% CO_2_ atmosphere. For microfluidic neuronal cell culture, 50 μL of hippocampal neuronal cells (1.5 × 10^5^ cells/mL) was seeded in the left compartment of the PDMS chamber.

### Immunocytochemistry (ICC)

Cells were fixed using 4% formaldehyde for 25 min and rinsed three times with PBS (100 mM, pH 7.4). The cells were then incubated in blocking solution, containing 4% BSA and 0.1% Triton X-100, dissolved in PBS for 30 min, followed by incubation in primary antibodies diluted in TBS (Tris-buffered saline, pH 7.4) containing 0.5% BSA and 0.1% Triton X-100, overnight at 4 °C. The samples were then washed three times with TBS and the fluorescent secondary antibodies were applied for 1 h at room temperature in TBS containing 0.5% BSA solution. The samples were washed again three times with TBS and once with ultrapure water, and stored in VECTASHIED Mounting Medium at −80 °C until microscopic examination. Fluorescence images were taken with a Zeiss LSM710 confocal laser scanning microscope equipped with ZEN 2009 software at the National Centre for Inter-university Research Facilities (NCIRF) of Seoul National University.

### Image quantification and analysis

To reflect both the frequency of synaptogenesis and the intensity of the hemisynapses formed by the artificial dendrites, at least 30 beads were randomly chosen as regions of interest (ROI) from the brightfield (differential interference contrast, DIC) image by excluding somal regions per condition per experiment using ZEN 2009 software. The same-sized ROI with the similar neurite density and without the bead was set as a control. The ROI profile was applied to each fluorescence channel and the intensity (MFI) was collected by ZEN 2009 software. For the time-dependent experiments (Figs 3 and 4), the MFI values of each channel was separately normalized to the maximum MFI values of the respective marker proteins. For the time- and compartment-dependent experiment using micropatterned neurons (Fig. 5), the overall MFI values of VGluT1 and VGAT were first normalized to the ones of Syn I, followed by the individual normalization to the maximum MFI values of the respective marker proteins. All images were prepared for figures using Photoshop (Adobe).

### Statistics

All statistics were performed using Origin 8.0 software. For comparisons of MFI between multi-group datasets, we carried out one-way ANOVA with Bonferroni’s method. All data shown are mean ± SEM (standard error of the mean). In figures, statistical significance is indicated by n.s. for p > 0.05, *for 0.05 > p > 0.01, **for 0.01 > p > 0.001, and ***for p > 0.001.

## Additional Information

**How to cite this article**: Kim, E. J. *et al.* Robust Type-specific Hemisynapses Induced by Artificial Dendrites. *Sci. Rep.* **6**, 24210; doi: 10.1038/srep24210 (2016).

## Supplementary Material

Supplementary Information

## References

[b1] Dalva M. B., McClelland A. C. & Kayser M. S. Cell adhesion molecules: signalling functions at the synapse. Nat. Rev. Neurosci. 8, 206–220, 10.1038/Nrn2075 (2007). 17299456 PMC4756920

[b2] Hu H., Gan J. & Jonas P. Interneurons. Fast-spiking, parvalbumin^+^ GABAergic interneurons: from cellular design to microcircuit function. Science 345, 1255263, 10.1126/science.1255263 (2014). 25082707

[b3] Normann R. A. Technology Insight: future neuroprosthetic therapies for disorders of the nervous system. Nature Clinical Practice Neurology 3, 444–452, 10.1038/Ncpneuro0556 (2007). 17671522

[b4] Orive G., Anitua E., Pedraz J. L. & Emerich D. F. Biomaterials for promoting brain protection, repair and regeneration. Nat. Rev. Neurosci. 10, 682–U647, 10.1038/Nrn2685 (2009). 19654582

[b5] Dean C. *et al.* Neurexin mediates the assembly of presynaptic terminals. Nat. Neurosci. 6, 708–716, 10.1038/Nn1074 (2003). 12796785 PMC1646425

[b6] Baksh M. M. *et al.* Neuronal activation by GPI-linked neuroligin-1 displayed in synthetic lipid bilayer membranes. Langmuir 21, 10693–10698, 10.1021/La051243d (2005). 16262338 PMC1448170

[b7] Czondor K. *et al.* Micropatterned substrates coated with neuronal adhesion molecules for high-content study of synapse formation. Nat. Commun. 4, 10.1038/Ncomms3252 (2013). 23934334

[b8] Gopalakrishnan G. *et al.* Lipid Bilayer Membrane-Triggered Presynaptic Vesicle Assembly. ACS Chem. Neurosci. 1, 86–94, 10.1021/Cn900011n (2010). 22778819 PMC3368651

[b9] Lucido A. L., Gopalakrishnan G., Yam P. T., Colman D. R. & Lennox R. B. Isolation of Functional Presynaptic Complexes from CNS Neurons: A Cell-Free Preparation for the Study of Presynaptic Compartments *In vitro*. ACS Chem. Neurosci. 1, 535–541, 10.1021/Cn100048z (2010). 22777142 PMC3368679

[b10] Lucido A. L. *et al.* Rapid assembly of functional presynaptic boutons triggered by adhesive contacts. J. Neurosci. 29, 12449–12466, 10.1523/Jneurosci.1381-09.2009 (2009). 19812321 PMC3849729

[b11] Sudhof T. C. Neuroligins and neurexins link synaptic function to cognitive disease. Nature 455, 903–911, 10.1038/Nature07456 (2008). 18923512 PMC2673233

[b12] Chih B., Engelman H. & Scheiffele P. Control of excitatory and inhibitory synapse formation by neuroligins. Science 307, 1324–1328, 10.1126/science.1107470 (2005). 15681343

[b13] Chih B., Gollan L. & Scheiffele P. Alternative splicing controls selective trans-synaptic interactions of the neuroligin-neurexin complex. Neuron 51, 171–178, 10.1016/j.neuron.2006.06.005 (2006). 16846852

[b14] Song J. Y., Ichtchenko K., Sudhof T. C. & Brose N. Neuroligin 1 is a postsynaptic cell-adhesion molecule of excitatory synapses. Proc. Natl. Acad. Sci. USA 96, 1100–1105, 10.1073/pnas.96.3.1100 (1999). 9927700 PMC15357

[b15] Giannone G. *et al.* Neurexin-1β Binding to Neuroligin-1 Triggers the Preferential Recruitment of PSD-95 versus Gephyrin through Tyrosine Phosphorylation of Neuroligin-1. Cell Reports 3, 1996–2007, doi: http://dx.doi.org/10.1016/j.celrep.2013.05.013 (2013). 23770246 10.1016/j.celrep.2013.05.013

[b16] Missler M., Sudhof T. C. & Biederer T. Synaptic Cell Adhesion. Cold Spring Harb. Perspect. Biol. 4, 10.1101/cshperspect.a005694 (2012). PMC3312681 22278667

[b17] Takahashi H. *et al.* Selective control of inhibitory synapse development by Slitrk3-PTP delta trans-synaptic interaction. Nat. Neurosci. 15, 389–U214, 10.1038/Nn.3040 (2012). 22286174 PMC3288805

[b18] Yim Y. S. *et al.* Slitrks control excitatory and inhibitory synapse formation with LAR receptor protein tyrosine phosphatases. Proc. Natl. Acad. Sci. USA 110, 4057–4062, 10.1073/pnas.1209881110 (2013). 23345436 PMC3593915

[b19] Comoletti D. *et al.* Characterization of the interaction of a recombinant soluble neuroligin-1 with neurexin-1 beta. J. Biol. Chem. 278, 50497–50505, 10.1074/jbc.M306803200 (2003). 14522992

[b20] Arac D. *et al.* Structures of neuroligin-1 and the Neuroligin-l/Neurexin-1 beta complex reveal specificprotein-protein and protein-Ca^2+^ interactions. Neuron 56, 992–1003, 10.1016/j.neuron.2007.12.002 (2007). 18093522

[b21] Chen X., Liu H., Shim A. H. R., Focia P. J. & He X. Structural basis for synaptic adhesion mediated by neuroligin-neurexin interactions. Nat. Struct. Mol. Biol. 15, 50–56, 10.1038/Nsmb1350 (2008). 18084303 PMC2922956

[b22] Howarth M. & Ting A. Y. Imaging proteins in live mammalian cells with biotin ligase and monovalent streptavidin. Nat. Protoc. 3, 534–545, 10.1038/nprot.2008.20 (2008). 18323822 PMC2671200

[b23] Rao A., Cha E. M. & Craig A. M. Mismatched appositions of presynaptic and postsynaptic components in isolated hippocampal neurons. J. Neurosci. 20, 8344–8353 (2000). 11069941 10.1523/JNEUROSCI.20-22-08344.2000 PMC6773189

[b24] Brunig I., Suter A., Knuesel I., Luscher B. & Fritschy J. M. GABAergic terminals are required for postsynaptic clustering of dystrophin but not of GABA(A) receptors and gephyrin. J. Neurosci. 22, 4805–4813 (2002). 12077177 10.1523/JNEUROSCI.22-12-04805.2002 PMC6757720

[b25] Hennou S., Khalilov I., Diabira D., Ben-Ari Y. & Gozlan H. Early sequential formation of functional GABA(A) and glutamatergic synapses on CA1 interneurons of the rat foetal hippocampus. Eur. J. Neurosci. 16, 197–208, 10.1046/j.1460-9568.2002.02073.x (2002). 12169102

[b26] Ben-Ari Y., Gaiarsa J. L., Tyzio R. & Khazipov R. GABA: A pioneer transmitter that excites immature neurons and generates primitive oscillations. Physiol. Rev. 87, 1215–1284, 10.1152/physrev.00017.2006 (2007). 17928584

[b27] Anderson T. R., Shah P. A. & Benson D. L. Maturation of glutamatergic and GABAergic synapse composition in hippocampal neurons. Neuropharmacology 47, 694–705, 10.1016/j.neuropharm.2004.07.023 (2004). 15458841

[b28] Peixoto R. T. *et al.* Transsynaptic Signaling by Activity-Dependent Cleavage of Neuroligin-1. Neuron 76, 396–409, 10.1016/j.neuron.2012.07.006 (2012). 23083741 PMC3783515

[b29] Taylor A. M. *et al.* A microfluidic culture platform for CNS axonal injury, regeneration and transport. Nat. Methods 2, 599–605, 10.1038/Nmeth777 (2005). 16094385 PMC1558906

[b30] Taylor A. M., Dieterich D. C., Ito H. T., Kim S. A. & Schuman E. M. Microfluidic Local Perfusion Chambers for the Visualization and Manipulation of Synapses. Neuron 66, 57–68, 10.1016/j.neuron.2010.03.022 (2010). 20399729 PMC2879052

[b31] Um J. W. *et al.* Structural basis for LAR-RPTP/Slitrk complex-mediated synaptic adhesion. Nat. Commun. 5, 10.1038/ncomms6423 (2014). 25394468

[b32] Kallberg M. *et al.* Template-based protein structure modeling using the RaptorX web server. Nat. Protoc. 7, 1511–1522, 10.1038/nprot.2012.085 (2012). 22814390 PMC4730388

[b33] Marvin J. S. *et al.* An optimized fluorescent probe for visualizing glutamate neurotransmission. Nat. Methods 10, 162–170, 10.1038/Nmeth.2333 (2013). 23314171 PMC4469972

