# Corrigendum: Robust Type-specific Hemisynapses Induced by Artificial Dendrites

**DOI:** 10.1038/srep28123

**Published:** 2016-06-20

**Authors:** Eun Joong Kim, Chang Su Jeon, Soo Youn Lee, Inseong Hwang, Taek Dong Chung

Scientific Reports 6: Article number: 2421010.1038/srep24210; published online: 04132016; updated: 06202016

In the original version of this Article, an additional affiliation for Taek Dong Chung was omitted. The correct affiliation is listed below:

Advanced Institutes of Convergence Technology, Suwon-Si, Gyeonggi-do 16229, Korea.

This error has now been corrected in the PDF and HTML versions of the Article.

