# Supplementary material for: Robust Type-specific Hemisynapses Induced by Artificial Dendrites
Source: Sci Rep. 2016 Apr 13;6:24210. doi: 10.1038/srep24210 (PMC4829863; doi:10.1038/srep24210)
Supplement: Supplementary Information [file srep24210-s1.pdf]

Supplementary Information

Robust Type-specific Hemisynapses Induced by Artificial Dendrites

Eun Joong Kim†, Chang Su Jeon†, Soo Youn Lee, Inseong Hwang* & Taek Dong Chung*

Department of Chemistry, Seoul National University, Seoul, 08826, Korea
*inseong@snu.ac.kr, tdchung@snu.ac.kr
†these authors contributed equally to this work

Table S1. Primers used for the generation of protein NL1-R. Sequences underlined are crossover area for the staggered PCR.

Primer name	DNA oligomer sequence from 5' end (length)
N38_KpnI-AP-1F	ggcggt ***GGTACC***TCATCTGCATAATCTCAATGACATT GGCGGCGGCAGCGGCGGAGGCAGCGAGGG (66)
N38-2_KpnI-GS-F	ggcggt ***GGTACC***GGCAGCGGCGGAGGCAGCGAGGG (35)
N39_AP-2R	GCCCTCGCTGCCGCCTCCGCTGCCGCCTCCGCTGCCTCCGCCCTCGCTGCCTCCGCCGCT (60)
N40_AP-3F	GCGGAGGCGGCAGCGAGGGCGGAGGCAGCGGCGGCGGCCTGAACGACATCTTCGAGGCCC (60)
N41_AP-NotI-4R	ggcagc ***GCGGCCGC***TTACTCGTGCCACTCGATCTTCTGGGCCTCGAAGATGTCGTTC (57)
N50_AP-PvuI-SalI-3F	GCGGAGGCGGCAGCGAGGGC ***CGATCG*** GGT ***GTCGAC*** GGCCTGAACGACATCTTCGAGGCCC (60)
N43_H8-PvuI-F	***CG***CACCATCACCACCACCACCATCACCGAT (30)
N44_H8-PvuI-R	CGGTGATGGTGGTGGTGGTGATGGTG***CGAT*** (30)
N49_PvuI-H8-TagRFP-F	ggc ***CGATCG***CACCATCACCACCACCACCATCACATGGTGTCTAAGGGCGAAGAG (54)
N48_EK-SalI-R	gccacc ***GTCGAC***CTTGTCGTCGTCGTCCTTGTACAGCTCGTCCATGC (47)
N55_NL2-BamHI-F	GTGTGGTCCACAGG ***GGATCC*** (20)
N56_NL2-631-639-R	TCTGTGTGCAGGTTGTGCAGGTG (23)
N74-2_Slitrk3-Ecto-KpnI-R	accgcc ***GGTACC***GCCCCCAGGAGGAGAAAACTC (33)
N83_pGW1-HindIII-F	CACCGTCCTTGACACG***AAGC*** (20)
N86_NotI-IRES-F	ct***GCGGCCGC***cgagcatagtcatctagcacaat TCCGCCCCTCTCCCTCCCCCCCCCCTAA (61)
N87_BirA-ER-NotI-R	ga***GCGGCCGC***TCACAGCTCGTCCTTTGAACCcccagatccagatgtagacccTTTTTCTGCACTACGCAGGG (72)
N103_NL2-631-639-F	CTGCACAACCTGCACACAGAGGGCGGCGGCAGCGG (35)
N104_SalI-AP-R	CGAAGATGTCGTTCAGGCC***G*** (20)
N106_SL3-shortGS-F	***C***GGCAGCGGCGGAGGCAGCGAGGGC***CGAT*** (29)
N107_SL3-shortGS-R	***CG***GCCCTCGCTGCCTCCGCCGCTGCCG***GTAC*** (31)

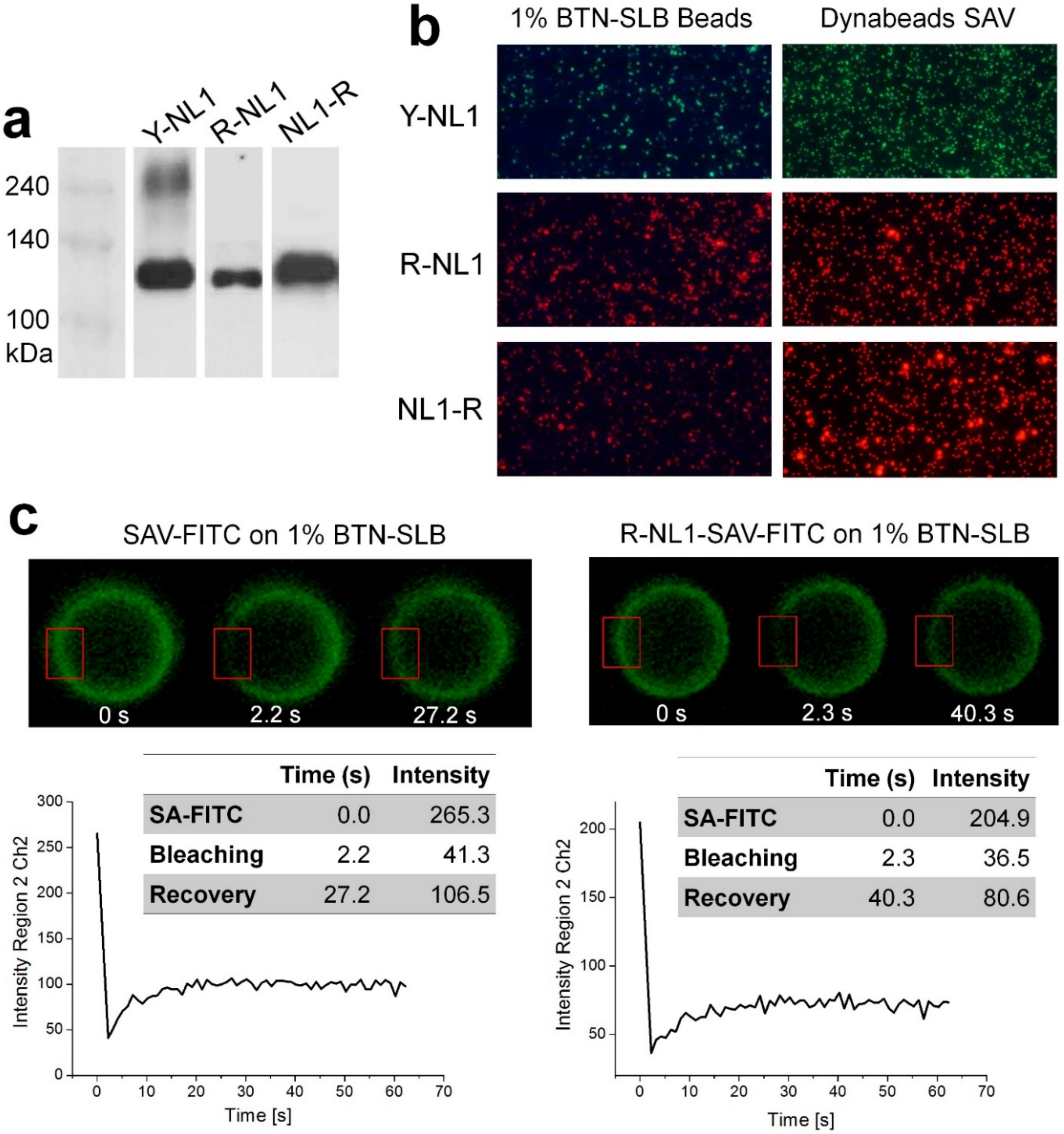

Supplementary Fig. S1. (a) Western blot profiles for the column-purified and biotinylated NL1 ectodomains. (b) The NL1 derivatives were immobilized either on supported lipid bilayer (SLB) or Sav-coated Dynabeads. (c) The membrane fluidity of bare and protein-loaded SLB was verified by fluorescence reactivation after photobleaching (FRAP) assay. Biotin-Cap-PE was mixed with egg PC as 1% (w/w) concentration, reconstituted on silica microbeads as SLB, treated with 170 nM FITC (fluorescein isothiocyanate)-coupled SAV before photobleaching within the red rectangular regions (left). After loading with R-NL1 the recovery time slightly increased (right). Left microscope images, before photobleaching; centre images, right after the photobleaching; right images, after fluorescence recovery.

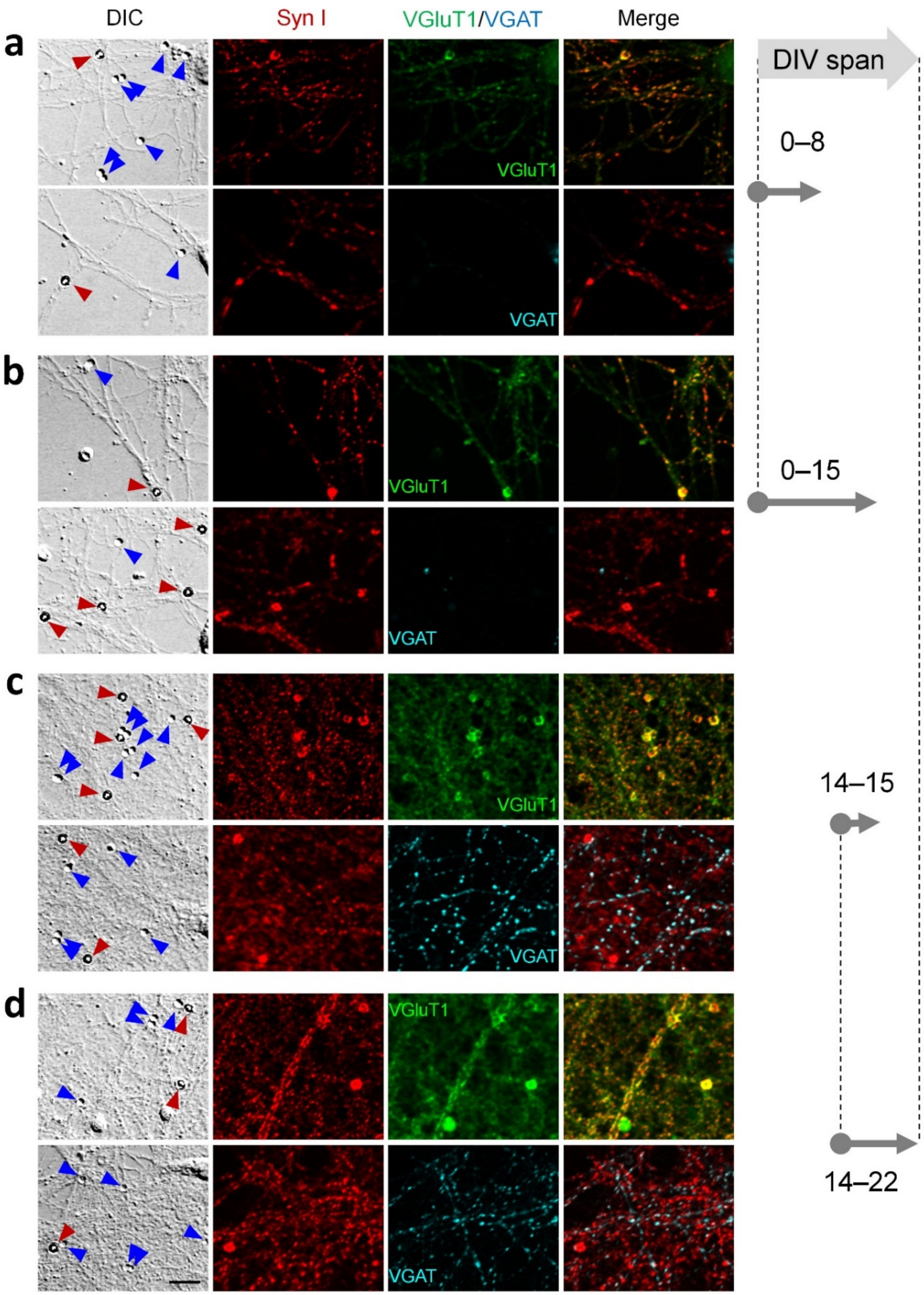
DIC
Syn I
VGluT1/VGAT
Merge
a
b
c
d
DIV span
0–8
0–15
14–15
14–22
VGluT1
VGAT
VGluT1
VGAT
VGluT1
VGAT
VGluT1
VGAT

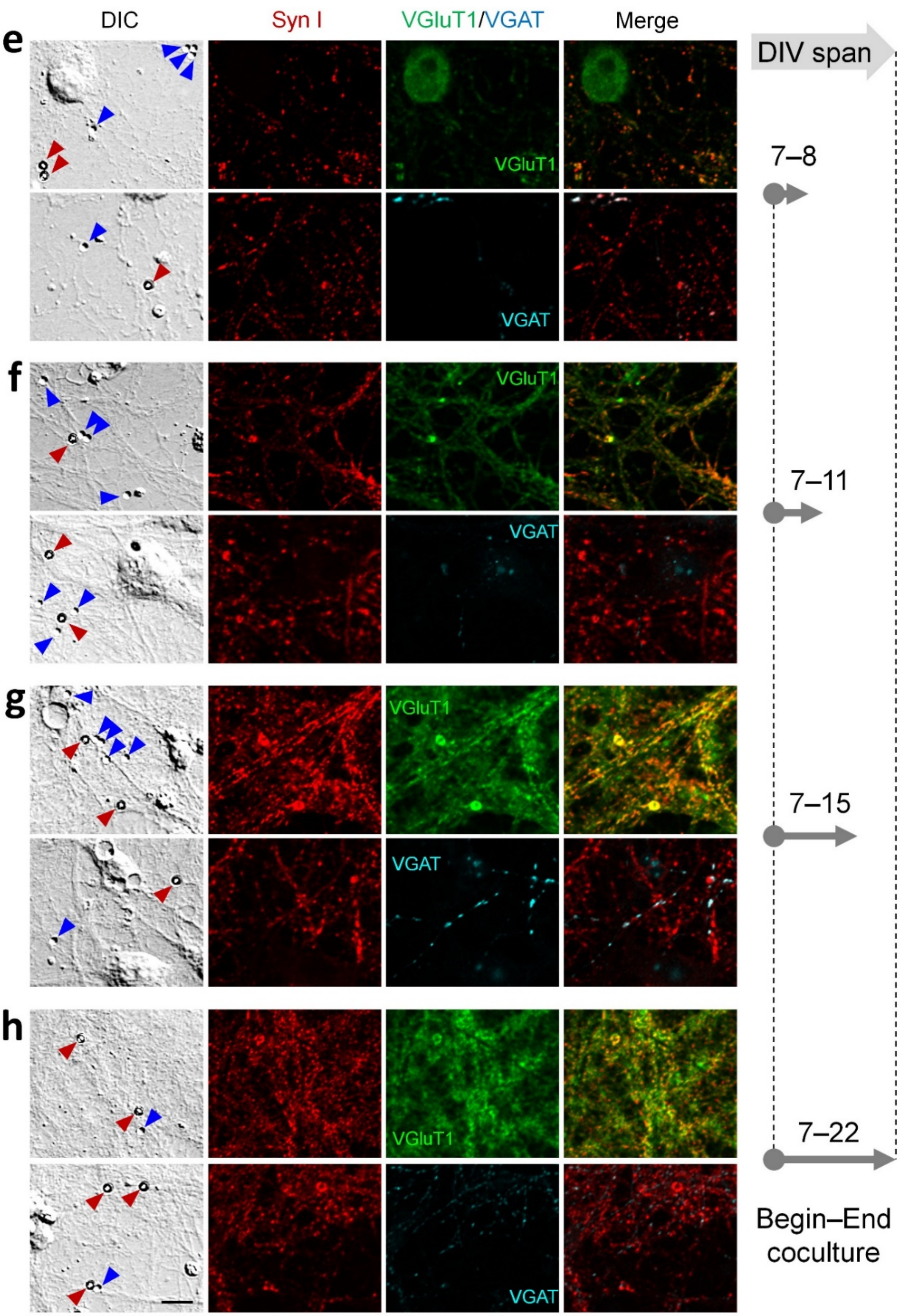

Supplementary Fig. S2. NL1-R induced the formation of glutamatergic presynaptic boutons regardless of the developmental stages of cultured neurons and the incubation period. The PDK and NL1-R beads were seeded on DIV0 and incubated for 8 d (a) and 15 d (b). The beads seeded on DIV14 were incubated for 1 d (c) and 8 d (d). When seeded on DIV7, the incubation was continued for 1 d (e), 4 d (f), 8 d (g), and 15 d (h). The cells were doubly stained either by anti-synapsin I (Syn I, red) and VGluT1 (green) (upper panels) or anti-Syn I and anti-VGAT (cyan) (lower panels). NL1-R bead, red arrowhead = 2.8 μm; PDK bead, blue arrowhead = 2.6 μm in diameter. Only the representative beads selected for the ROIs are shown. Scale bar = 10 μm.

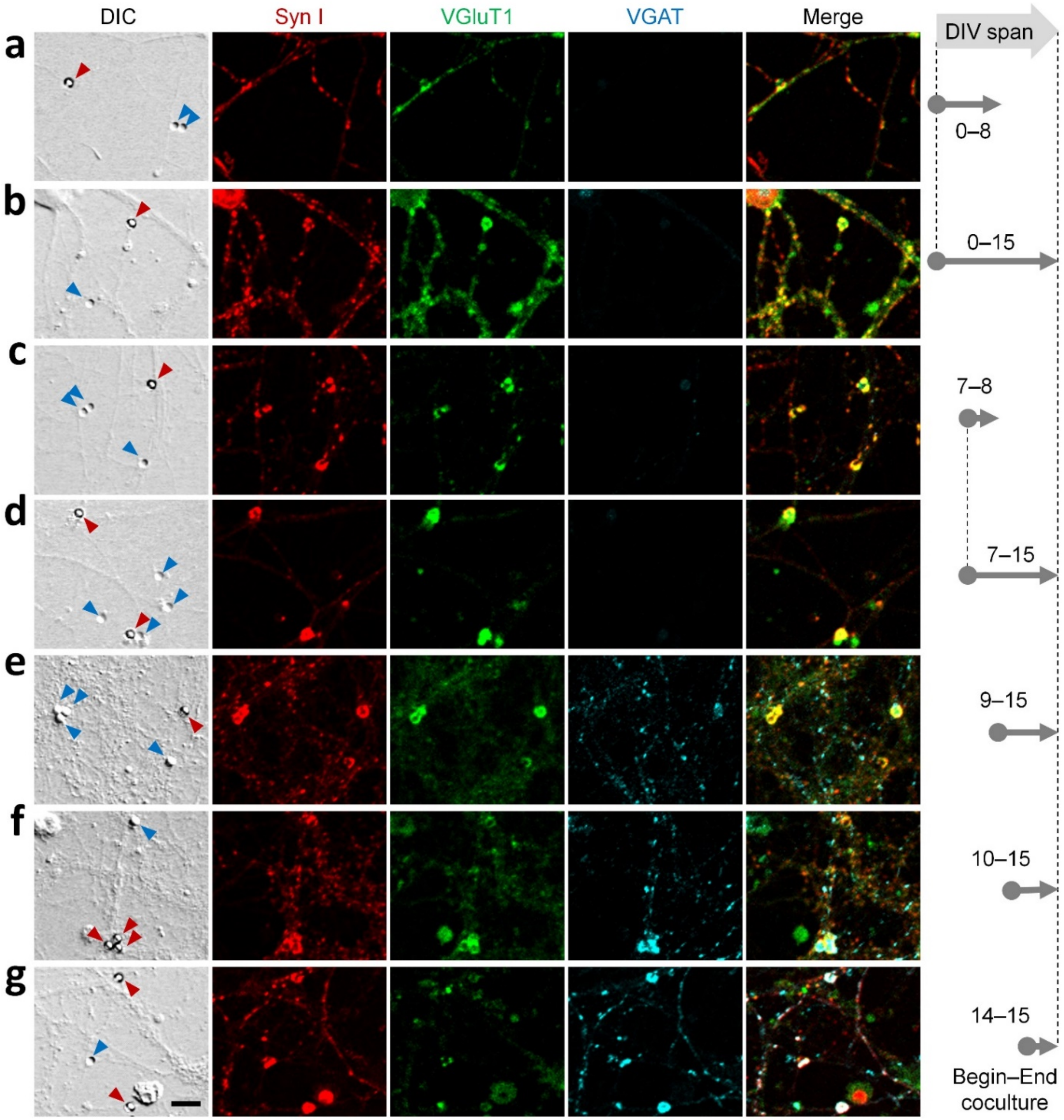
DIC
Syn I
VGluT1
VGAT
Merge
DIV span
a
b
c
d
e
f
g
0–8
0–15
7–8
7–15
9–15
10–15
14–15
Begin–End coculture

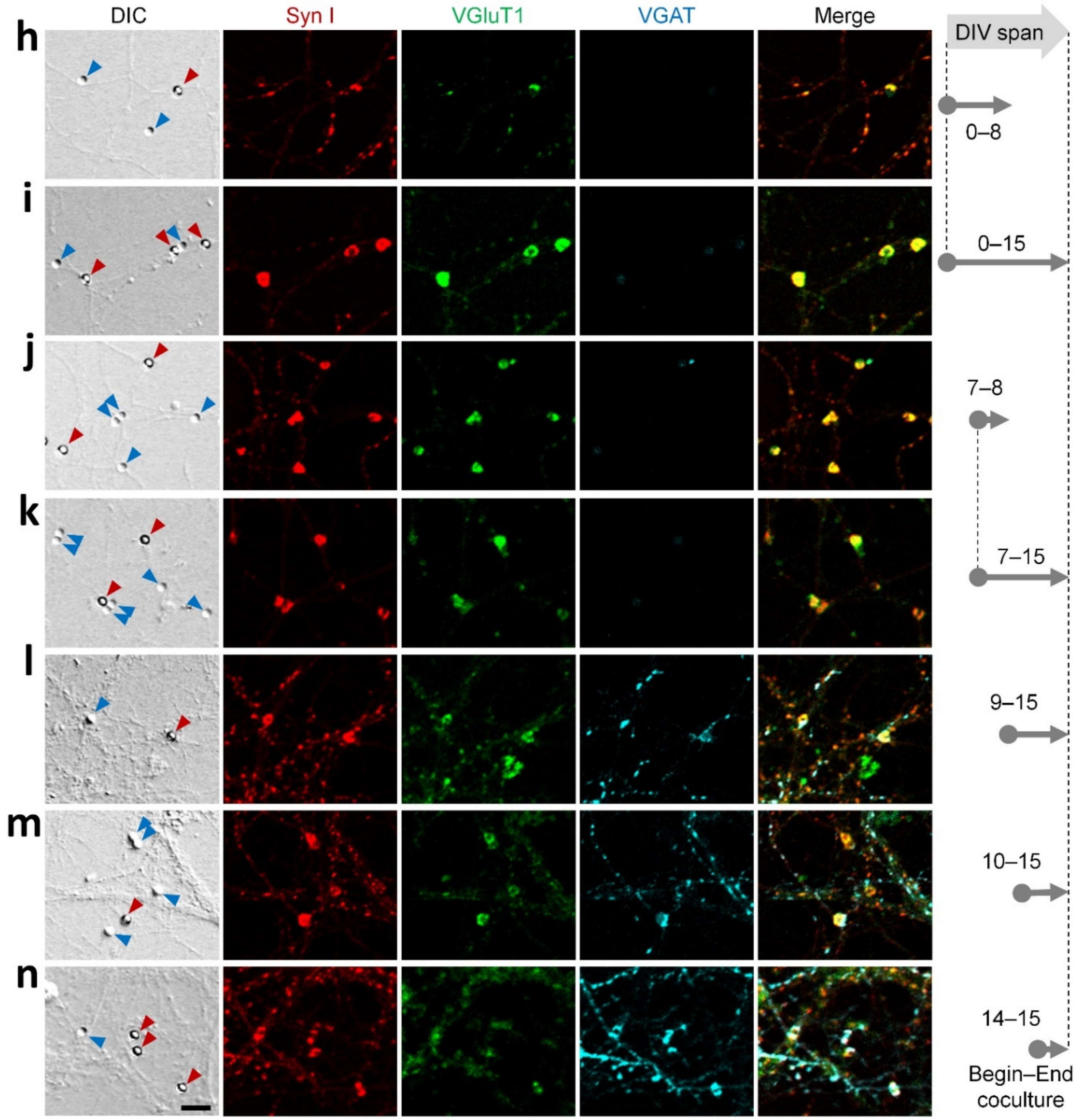

Supplementary Fig. S3. The NL2-R and SL3-R beads were seeded and analysed for the aggregation of Syn I (red), VGluT1 (green), and VGAT (cyan) on DIV0-8 (a and h), DIV0-15 (b and i), DIV7-8 (c and j), DIV7-15 (d and k), DIV9-15 (e and l), DIV10-15 (f and m), and DIV14-15 (g and n), respectively. NL1-R bead, red arrowhead = 2.8 μm; PDK bead, blue arrowhead = 2.6 μm in diameter. Only the representative beads selected for the ROIs are shown. Scale bar = 10 μm.

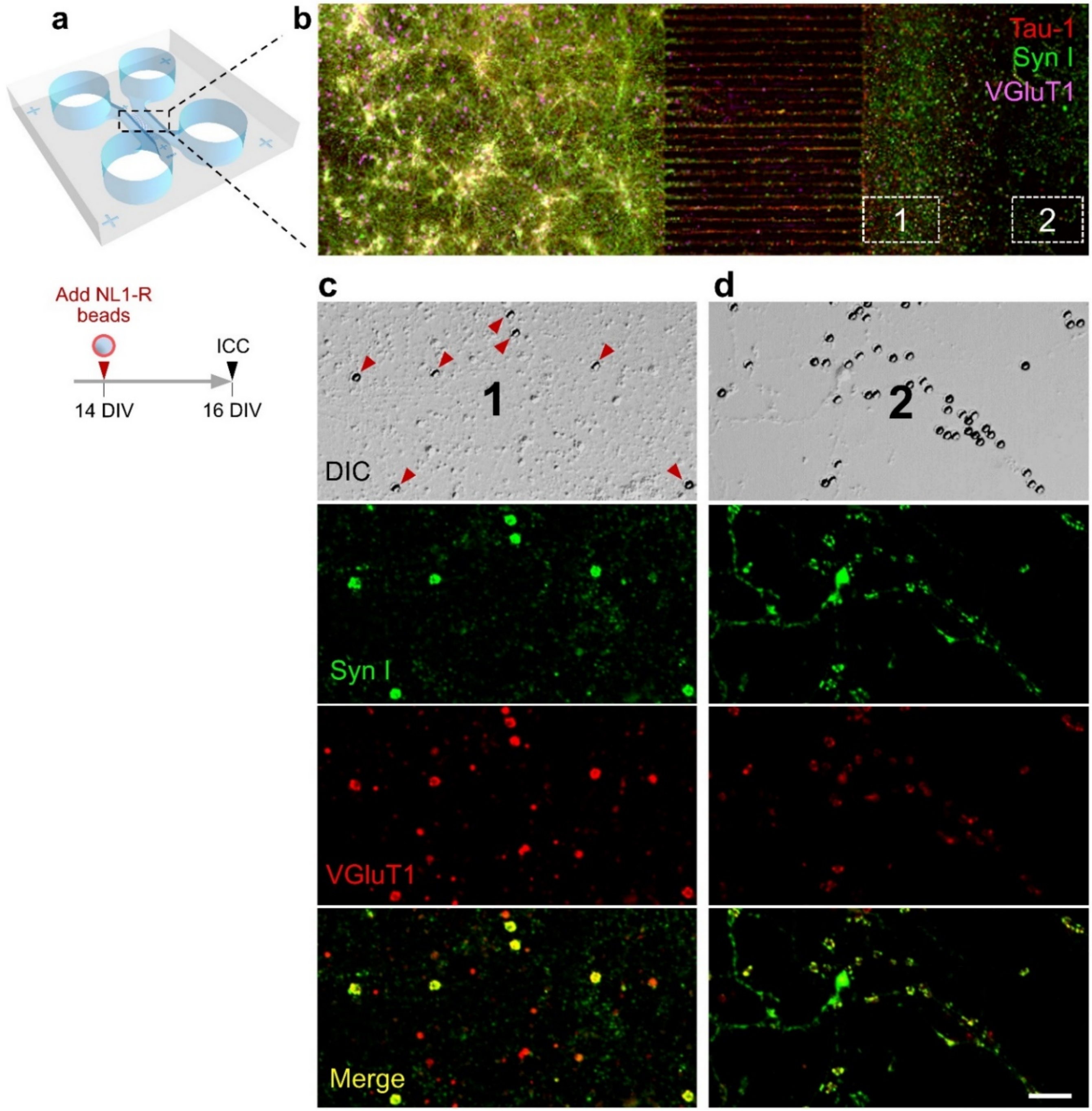

Supplementary Fig. S4. Axons isolated by compartmentalized culture chamber can elicit glutamatergic presynaptic differentiation. (a) On the axonal compartment the NL1-R displaying artificial dendritic beads were seeded on DIV14 and incubated for 2 d. (b) Immunoreactivity of Tau-1, Syn I, and VGlut1. Tau-1 is an axonal marker protein. (c) The artificial dendrites near the microchannel exits exhibit stronger fluorescent puncta (red arrowheads), due to the longer co-culture period, than the ones on outgrowing axonal terminals (d). Scale bar = 15 μm.

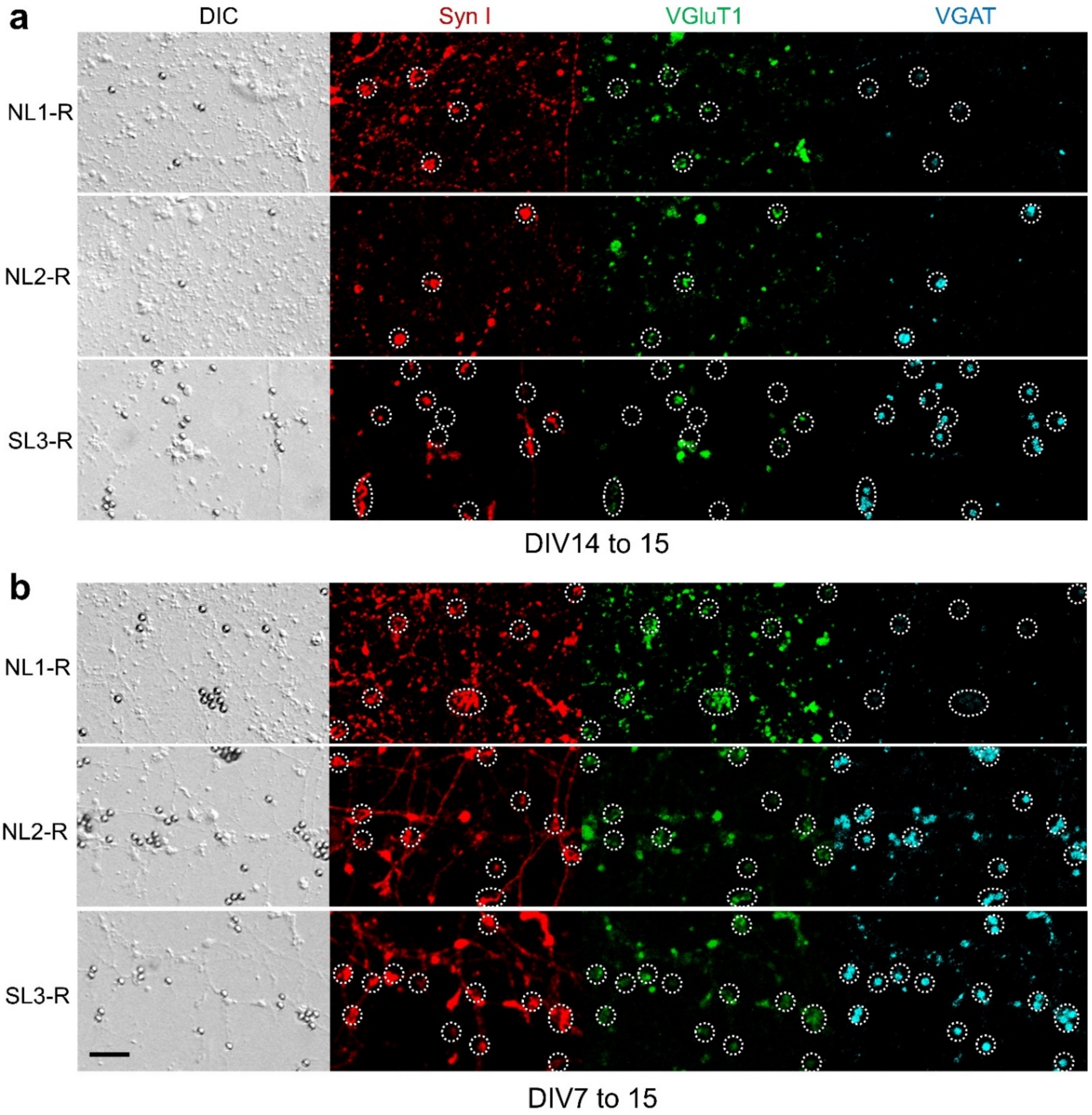

Supplementary Fig. S5. Synaptic specificity was maintained when the artificial dendrites of NL1-R, NL2-R and SL3-R were seeded on DIV14 and incubated for 1 d (a) and were seeded on DIV7 and incubated for 8 d (b) with axonal compartment. Representative bead-containing regions are shown as broken line circles. Scale bar = 15 μm.

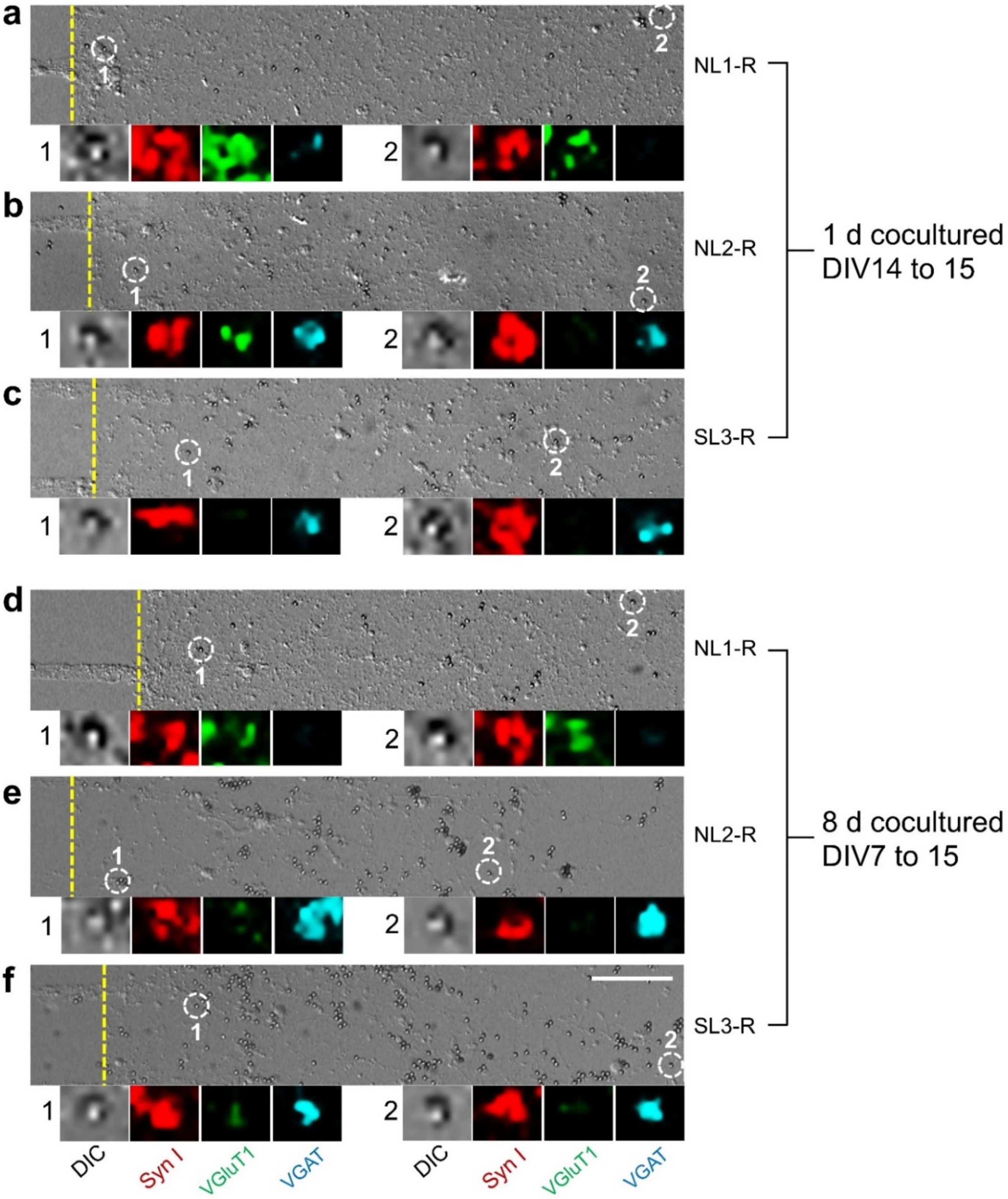

Supplementary Fig. S6. Synaptic specificity induced by the artificial dendrites showed little difference at near (a-c) and far from (d-f) the microchannels regardless of the seed timing of the dendritic beads. The end of the microchannels is indicated by yellow broken lines.
